# First-line treatment of patients with HER2-positive metastatic gastric and gastroesophageal junction cancer

**DOI:** 10.17305/bjbms.2021.7069

**Published:** 2022-04-18

**Authors:** Selin Aktürk Esen, Yakup Ergun, Cihan Erol, Rukiye Arikan, Muhammed Muhiddin Er, Muhammed Mustafa Atci, Atakan Topçu, Gökhan Uçar, Baran Akagündüz, Musa Bariş Aykan, Miraç Özen, Naziyet Köse Baytemur, Melike Özçelik, Elif Şahin, Denizcan Güven, Serkan Menekşe, Naziye Ak, Fatih Teker, Engin Kut, Teoman Şakalar, Özkan Alan, Turgut Kaçan, Nazim Serdar Turhal, Saadettin Kiliçkap, Sema Türker, Mehmet Ali Nahit Şendur, Osman Köstek, Mustafa Karaağaç, Abdullah Sakin, Haci Mehmet Türk, Dilek Çağlayan, Şener Cihan, Yusuf Açikgöz, Doğan Uncu

**Affiliations:** 1 Department of Medical Oncology, Ankara City Hospital, Ankara, Türkiye; 2 Department of Medical Oncology, Batman Training and Research Hospital, Batman, Türkiye; 3 Department of Medical Oncology, Faculty of Medicine, Marmara University, Istanbul, Türkiye; 4 Department of Medical Oncology, Meram Faculty of Medicine, Necmettin Erbakan University, Konya, Türkiye; 5 Department of Medical Oncology, Prof. Dr. Cemil Taşcioğlu City Hospital, Istanbul, Türkiye; 6 Department of Medical Oncology, Faculty of Medicine, Bezmialem Vakif University, Istanbul, Türkiye; 7 Department of Medical Oncology, Erzincan Binali Yildirim University, Erzincan, Türkiye; 8 Department of Medical Oncology, Gulhane Medical Faculty, Health Sciences University, Ankara, Türkiye; 9 Department of Medical Oncology, Faculty of Medicine, Sakarya University Sakarya, Türkiye; 10 Department of Medical Oncology, Memorial Ankara Hospital, Ankara, Türkiye; 11 Department of Medical Oncology, Ümraniye Training and Research Hospital, İstanbul, Türkiye; 12 Department of Medical Oncology, Faculty of Medicine, Kocaeli University, Kocaeli, Türkiye; 13 Department of Medical Oncology, Faculty of Medicine, Hacettepe University, Ankara, Türkiye; 14 Department of Medical Oncology, Manisa City Hospital, Manisa, Türkiye; 15 Department of Medical Oncology, Yozgat City Hospital, Yozgat, Türkiye; 16 Department of Medical Oncology, Faculty of Medicine, Gaziantep University, Gaziantep, Türkiye; 17 Department of Medical Oncology, Kahramanmaraş Necip Fazil City Hospital, Kahramanmaraş, Türkiye; 18 Department of Medical Oncology, Tekirdağ İsmail Fehmi Cumalioğlu City Hospital, Tekirdağ, Türkiye; 19 Department of Medical Oncology, Yüksek İhtisas Training and Research Hospital, Health Sciences University Bursa, Bursa, Türkiye; 20 Department of Medical Oncology, Anadolu Health Center, İstanbul, Türkiye; 21 Department of Medical Oncology, Liv Hospital Ankara, Faculty of Medicine, İstinye University, Ankara, Türkiye; 22 Department of Internal Medicine, Faculty of Medicine, Bahcesehir University, Istanbul, Türkiye

**Keywords:** CF, FOLFOX, gastric cancer, gastroesophageal junction cancer, trastuzumab

## Abstract

Fluoropyrimidine+cisplatin/oxaliplatin+trastuzumab therapy is recommended for the first-line treatment of human epidermal growth factor receptor 2 (HER2)-positive metastatic gastric adenocarcinoma. However, there is no comprehensive study on which platinum-based treatment should be preferred. This study aimed to compare the treatment response and survival characteristics of patients with HER2-positive metastatic gastric or gastroesophageal junction (GEJ) cancer who received fluorouracil, oxaliplatin, and leucovorin (mFOLFOX)+trastuzumab or cisplatin and fluorouracil (CF)+trastuzumab as first-line therapy. It was a multicenter, retrospective study of the Turkish Oncology Group, which included 243 patients from 21 oncology centers. There were 113 patients in the mFOLFOX+trastuzumab arm and 130 patients in the CF+trastuzumab arm. The median age was 62 years in the mFOLFOX+trastuzumab arm and 61 years in the CF+trastuzumab arm (*p* = 0.495). About 81.4% of patients in the mFOLFOX+trastuzumab arm and 83.1% in the CF+trastuzumab arm had gastric tumor localization (*p* = 0.735). The median progression-free survival (PFS) was significantly higher in the mFOLFOX+trastuzumab arm (9.4 months vs. 7.3 months, *p* = 0.024). The median overall survival (OS) was similar in both groups (18.4 months vs. 15.1 months, *p* = 0.640). Maintenance trastuzumab was continued after chemotherapy in 101 patients. In this subgroup, the median OS was 23.3 months and the median PFS was 13.3 months. In conclusion, mFOLFOX+trastuzumab is similar to CF+trastuzumab in terms of the median OS, but it is more effective in terms of the median PFS in the first-line treatment of HER2-positive metastatic gastric and GEJ cancer. The choice of treatment should be made by considering the prominent toxicity findings of the chemotherapy regimens.

## INTRODUCTION

Although the incidence of gastric cancer has decreased in Western Europe and the United States, it still remains a major health problem around the world [1-3]. It is the fifth most common cancer worldwide and ranks third in cancer-related deaths [4].

Human epidermal growth factor receptor 2 (HER2) belongs to the epidermal growth factor receptor family, which plays an important role in the activation of signal transduction pathways that control epithelial cell growth, differentiation [5,6], and angiogenesis [7]. Overexpression of the HER2 and/or HER2 gene amplification can be detected in many solid tumors. In 1998, a significant survival advantage has been achieved with the use of trastuzumab, an anti-HER2-targeted agent, in the treatment of highly aggressive HER2-positive breast cancer [8,9]. The rate of HER2 positivity in gastric adenocarcinoma patients varies between 12% and 23% [10-14]. Unlike in breast cancer, the importance of HER2 overexpression or amplification in gastric cancer prognosis is unclear. While some studies [12,15] have claimed that HER2 positivity has no prognostic significance in gastric cancer patients, some studies have shown that it is related to poor prognosis [10,16].

The NCCN guideline recommendation in the treatment of HER2-positive metastatic gastric adenocarcinoma is fluoropyrimidine+cisplatin/oxaliplatin+trastuzumab therapy. In the NCCN guidelines, the combination of fluoropyrimidine+cisplatin+trastuzumab is recommended as category 1 [17]. The TOGA phase 3 trial [18] compared chemotherapy (capecitabine+cisplatin or fluorouracil+cisplatin) versus chemotherapy+trastuzumab in the first-line therapy of patient’s metastatic gastric or gastroesophageal junction (GEJ) cancer. Significant overall survival (OS) and progression-free survival (PFS) were achieved in the chemotherapy+trastuzumab arm. With the TOGA trial, the addition of trastuzumab to chemotherapy has become the standard treatment approach for patients with HER2-positive metastatic gastric or GEJ. In the phase 2 HERXO trial [19] in 45 patients, trastuzumab+capecitabine and oxaliplatin (XELOX) were administered as first-line therapy for HER2-positive metastatic gastric or GEJ cancer. In this study, it was emphasized that other platinum-based therapies with the addition of trastuzumab could be an alternative to cisplatin in the first-line treatment of metastatic gastric or GEJ cancer.

In the literature, studies with a very small number of patients were found comparing trastuzumab in combination with cisplatin or oxaliplatin as first-line therapy in patients with metastatic gastric or GEJ cancer. This study aimed to compare the real-life data of cisplatin and fluorouracil (CF) in combination with trastuzumab and fluorouracil, oxaliplatin, and leucovorin (mFOLFOX) in combination with trastuzumab in the first-line treatment in patients with metastatic gastric or GEJ cancer.

## MATERIALS AND METHODS

This study was designed and conducted as a multicenter, retrospective study of the Turkish Oncology Group. Study included 243 patients from 21 oncology centers. The inclusion criteria were as follows: (1) having been histopathologically diagnosed with HER2-positive gastric or GEJ adenocarcinoma, (2) being aged 18 years and over ; (3) having Eastern Cooperative Oncology Group Performance Status (ECOG PS) of 0, 1, or 2; and (4) not having received treatment in the metastatic phase. HER2 was evaluated by immunohistochemistry from primary or metastatic tumor tissue, and those evaluated with a score of 2 or 3 underwent fluorescent *in situ* hybridization (FISH). Tumor cell clusters with a weak to moderate complete, basolateral, or lateral membranous reactivity, irrespective of the percentage of tumor cells stained in the biopsy specimen, and weak to moderate complete, basolateral, or lateral membranous reactivity in ≥10% of the tumor cells in the operation specimen were accepted as a HER2 score of 2. Tumor cell clusters with a strong complete, basolateral, or lateral membranous reactivity, irrespective of the percentage of tumor cells stained in the biopsy specimen, and strong complete, basolateral, or lateral membranous reactivity in ≥10% of the tumor cells in the operation specimen were accepted as a HER2 score of 3. The tumor was considered HER2-positive if the HER2 gene amplification was detected by FISH (HER2/CEP17 ratio ≥2).

Clinicopathological features, radiological findings, treatments, and toxicity findings of the patients were scanned from their files. Chemotherapy regimens in combination with trastuzumab were CF (cisplatin 80 mg/m^2^ as intravenous [IV] infusion on day 1, fluorouracil 800 mg/m^2^ per day as continuous IV infusion on days 1-5 repeated every 3 weeks) and mFOLFOX (oxaliplatin 85 mg/m^2^, leucovorin 400 mg/m^2^ as IV infusion on day 1, followed by bolus 5-FU 400 mg/m^2^ IV infusion on day 1, and then 5-FU 2400 mg/m^2^ as a continuous IV infusion over 46 h starting on day 1, repeated every 14 days). A maximum of 6 cycles of CF chemotherapy regimen were given every 21 days. A maximum of 12 cycles of mFOLFOX chemotherapy regimen were administered every 14 days. Trastuzumab was administered at a dose of 8 mg/kg in the initial cycle and then at a dose of 6 mg/kg every 3 weeks with the CF regimen, and at a dose of 6 mg/kg in the initial cycle and then at a dose of 4 mg/kg every 2 weeks with the mFOLFOX regimen until disease progression or unacceptable toxicity occurred. In Turkey, trastuzumab is covered by reimbursement in combination with CF or FOLFOX chemotherapy in HER2 + metastatic gastric and GEJ cancer patients. Maintenance trastuzumab approval was obtained from the Turkish Drug and Medical Device Institution. RECIST 1.1 criteria were used to determine the response to the treatment.

OS was defined as the time interval from the time of onset of metastatic disease to death due to any reason or last follow-up. PFS was defined as the time interval from initiation of the treatment with trastuzumab to progression or death from any cause.

The study was approved by the Ankara City Hospital Ethics Committee under decision number E1/2174/2021 in compliance with the Helsinki Declaration.

### Statistical analysis

Continuous variables were presented as the median (minimum-maximum) and categorical variables were presented as percentages. Normality of the data was analyzed using the Kolmogorov–Smirnov and Shapiro–Wilk tests. The Pearson chi-square test was used to compare the categorical variables of the two groups, and the independent sample t-test or Mann–Whitney U-test was used to compare the continuous variables of the two groups. The Kaplan–Meier method was used for the survival analysis. *p* < 0.05 was considered statistically significant. IBM SPSS Statistics for Windows 24.0 (IBM Corp., Armonk, NY, USA) was used for the statistical analyses.

## RESULTS

A total of 243 patients were included in the study. There were 113 patients in the mFOLFOX+trastuzumab arm and 130 patients in the CF+trastuzumab arm. The median age was 62 (27-80) years in the mFOLFOX+trastuzumab arm and 61 (28-83) years in the CF+trastuzumab arm (*p* = 0.495). Tumor localization was gastric in 81.4% and GEJ 18.6% of the patients in the mFOLFOX+trastuzumab arm. In the CF+trastuzumab arm, 83.1% of the patients had gastric localization and 16.9% had GEJ localization. There was no difference between the two groups in terms of tumor localization (*p* = 0.735). 100 patients (88.5%) in the mFOLFOX+trastuzumab arm and 100 patients (76.9%) in the CF+trastuzumab arm had *de* *novo* metastases. Number of *de novo* metastatic patients was significantly higher in the mFOLFOX+trastuzumab arm (*p* = 0.018). There was no statistically significant difference between the two groups regarding the patients’ gender, ECOG-PS, and comorbidity (Table 1).

**TABLE 1:**
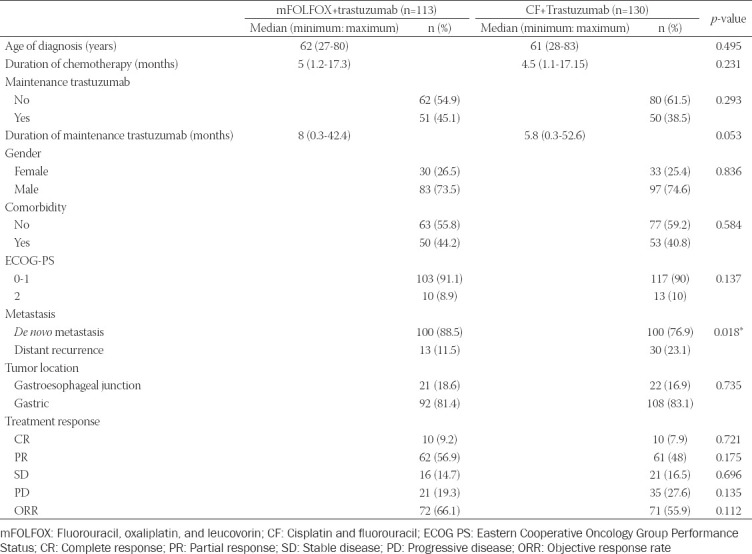
Clinical characteristics of the treatment groups

The median chemotherapy duration of the patients in the mFOLFOX+trastuzumab group was 5 months, and the median maintenance trastuzumab duration was 8 months. The median chemotherapy duration of the CF+trastuzumab group was 4.5 months, and the median maintenance trastuzumab duration was 5.8 months. There was no difference between the two groups in terms of chemotherapy or trastuzumab treatment duration (*p* = 0.231 and *p* = 0.053, respectively) (Table 1).

The treatment response results of a total of 236 patients were available. In the mFOLFOX+trastuzumab arm, 10 patients (9.2%) had complete response (CR), 62 (56.9%) had partial response (PR), 16 (14.7%) had stable disease (SD), and 21 (19.3%) had progressive disease (PD). In the CF+trastuzumab arm, 10 patients (7.9%) had CR, 61 (48%) PR, 21 (16.5%) had SD, and 35 (27.6%) had PD. The objective response rate (ORR) was obtained in 72 patients (66.1%) who received mFOLFOX+trastuzumab, while it was obtained in 71 patients (55.9%) who received CF+trastuzumab. There was no difference in the rates of CR, PR, SD, PD, and ORR between the two groups (*p* = 0.721, *p* = 0.175, *p* = 0.696, *p* = 0.135, and *p* = 0.112, respectively) (Table 1).

**FIGURE 1:**
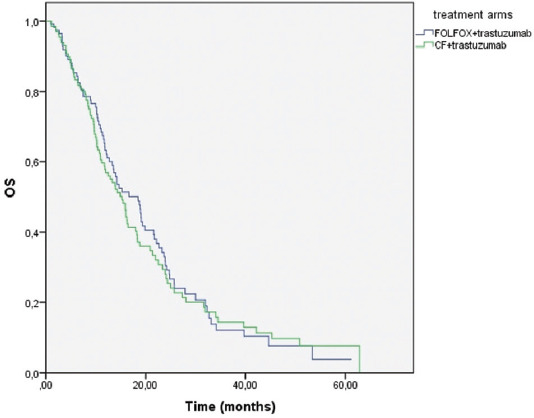
Kaplan–Meier analysis for OS of the treatment arms (mFOLFOX+trastuzumab vs. CF+trastuzumab). FOLFOX: Fluorouracil, oxaliplatin, and leucovorin; CF: Cisplatin and fluorouracil; OS: Overall survival.

**FIGURE 2:**
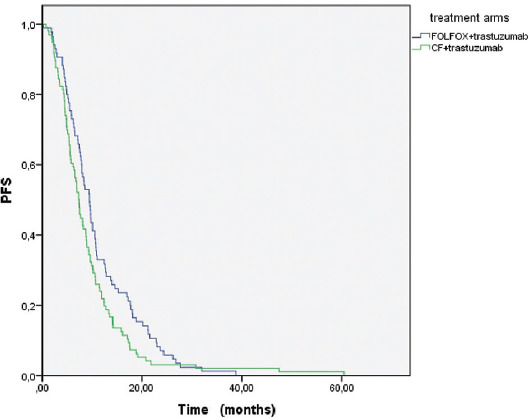
Kaplan–Meier analysis for PFS of the treatment arms (mFOLFOX+trastuzumab vs. CF+trastuzumab). FOLFOX: Fluorouracil, oxaliplatin, and leucovorin; CF: Cisplatin and fluorouracil; PFS: Progression-free survival.

The median OS was 18.4 (14-22.8) months in the mFOLFOX+trastuzumab arm and 15.1 (12.3-18) months in the CF+trastuzumab arm (*p* = 0.640) (Figure 1). The median PFS was statistically significantly higher in the mFOLFOX+trastuzumab arm than in the CF+trastuzumab arm (9.4 [8-10.9] months vs. 7.3 [6.4-8.2] months) (*p* = 0.024) (Figure 2).

At the end of the study, 34 (13.9%) patients were still on maintenance trastuzumab therapy, and 22 patients (9%) stopped receiving treatment voluntarily while on maintenance trastuzumab therapy. The reason for discontinuation of the trastuzumab treatment was disease progression in 169 patients (69.7%), toxicity in 8 patients (3.3%), and death from any cause in 10 patients (4.1%). Four of the patients who developed toxicity (decrease in left ventricular ejection fraction [LVEF] of grade 3 and above) were in the mFOLFOX+trastuzumab arm. Four of the patients who developed toxicity (1 patient due to trastuzumab anaphylaxis, and 3 patients due to a decrease in LVEF of grade 3 and above) were in the CF+trastuzumab arm. There were no deaths due to trastuzumab or chemotherapy toxicity.

**FIGURE 3:**
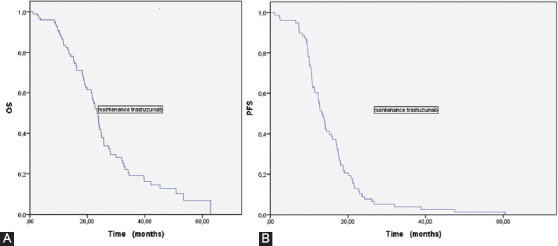
(A) Kaplan–Meier analysis for OS in patients receiving maintenance trastuzumab; (B) Kaplan–Meier analysis for PFS in patients receiving maintenance trastuzumab. OS: Overall survival; PFS: Progression-free survival.

There were 101 patients who continued maintenance trastuzumab after chemotherapy+trastuzumab. Patients who received maintenance trastuzumab were analyzed as a subgroup. The median age of these patients was 59 (28-76) years. Twenty-three (22.8%) patients were female and 78 (77.2%) were male. In patients receiving maintenance trastuzumab, 90.1% of the tumors were gastric localized and 9.9% were GEJ localized. There was *de novo* metastasis in 89.1% of the patients. Moreover, 50.5% of the patients received FOLFOX+trastuzumab and 49.5% received CF+trastuzumab. Thirteen (12.9%) patients developed CR, 70 (69.3%) developed PR, 14 (13.9%) developed SD, and 4 (4%) developed PD. The median OS was 23.3 (21.5-25.1) months (Figure 3A). The median PFS was 13.3 (11.6-14.9) months (Figure 3B).

## DISCUSSION

This retrospective observational study compared the real-life data of CF+trastuzumab versus mFOLFOX+trastuzumab as first-line therapy in patients with metastatic gastric and GEJ adenocarcinoma. To the best of our knowledge, this is the first study with a large number of patients to compare these two chemotherapy regimens combined with trastuzumab as first-line therapy in patients with metastatic gastric and GEJ adenocarcinoma. In this study, the median OS in the mFOLFOX+trastuzumab arm was 18.4 months. Although a 3.3-month OS benefit was detected in the mFOLFOX+trastuzumab arm, it did not reach statistical significance. However, the median PFS was significantly higher in the mFOLFOX+trastuzumab arm when compared to the CF+trastuzumab arm.

In the phase 3 TOGA trial [18] published in 2010, it was shown that patients with a higher expression of HER2 benefited more by the addition of trastuzumab. The median OS was 13.8 months in the chemotherapy+trastuzumab arm and 11.1 months in the chemotherapy arm (*p* = 0.0046). The median PFS was 6.7 months in the trastuzumab arm and 5.5 months in the chemotherapy arm (*p* = 0.0002). After this study, adding trastuzumab to chemotherapy became the standard treatment approach for HER2-positive gastric and GEJ patients, with a 26% reduction in mortality. In the TOGA trial, in the chemotherapy+trastuzumab arm, 5% of patients had CR, 42% had PR, 32% had SD, and 12% had PD. In that study, in the CF+trastuzumab arm, 7.9% of the patients had CR, 48% had PR, 16.5% had SD, and 27.6% had PD. In the study conducted herein, in the CF+trastuzumab arm, median the OS and median PFS were numerically higher when compared to the same treatment arm in the TOGA study. Moreover, the ORR seemed to be better in our study, which may have been because capecitabine was used instead of fluorouracil in addition to cisplatin in the TOGA trial. In the combination with trastuzumab+cisplatin, the capecitabine may have been less effective than the fluorouracil. Another important study on this subject is the phase 2 HERXO trial [19]. Forty-five patients with advanced or GEJ cancer received XELOX+trastuzumab as first-line therapy. The median OS was 13.8 months and the median PFS was 7.1 months in the HERXO trial. The tumor response rates were as follows: CR was 8.9%, PR was 37.8%, and SD was 31.1%. In the HERXO trial, it was emphasized that other platinum-based therapies with the addition of trastuzumab could be an alternative to cisplatin in the first-line treatment of metastatic gastric or GEJ cancer. There are also phase 2 studies investigating the efficacy of XELOX+trastuzumab conducted in different centers. In a phase 2 study conducted in Korea in 64 patients with chemotherapy-naive, locally advanced, unresectable or metastatic gastric or GEJ adenocarcinoma [20], the patients received a median of 10 cycles of capecitabine, 8 cycles of oxaliplatin, and 10 cycles of trastuzumab. The median OS was 21 months, median PFS was 9.8 months, and the treatment response rates were as follows: CR was 4%, PR was 64%, and SD was 21%. In the phase 2 study of Gong et al. [21], XELOX+trastuzumab was administered to 51 patients with unresectable, locally advanced or metastatic gastric/GEJ adenocarcinoma. The median OS was 19.5 months, median PFS was 9.2 months, and the treatment response rates were as follows: CR was 1.9%, PR was 64.7%, and SD was 19.6%. In these 3 above-mentioned phase 2 studies, the median OS ranged from 13.8 months to 21 months, and the median PFS ranged from 7.1 months to 9.8 months. However, these studies had relatively small patient numbers when compared to the number of patients in the FOLFOX+trastuzumab arm in the study conducted herein. In addition to metastatic patients, unresectable, locally advanced patients were also included in these studies. Our study included only patients with metastatic disease and the median OS and median PFS were better than those in the HERXO study and were similar to those in the other two phase 2 studies, which may suggest that oxaliplatin and fluorouracil work better than oxaliplatin and capecitabine in combination with trastuzumab. Another study supporting this hypothesis was conducted by Jimenez-Fonseca et al. [22], conducted on patients with advanced distal esophageal, GEJ, and gastric adenocarcinomas, in which 158 patients (26.5%) received XELOX+trastuzumab, 86 (14.4%) received FOLFOX+trastuzumab, and 81 (13.6%) received CF+trastuzumab treatment. In the FOLFOX+trastuzumab arm, the median PFS was 9.6 months, and the median OS was 16.5 months; in the CF+trastuzumab arm, the median PFS was 7.8 months, and the median OS was 13.7 months; and in the XELOX+trastuzumab arm, the median PFS was 7.7 months, and the median OS was 13.7 months. In this study, median PFS and median OS in the FOLFOX+trastuzumab arm were better than in the XELOX+trastuzumab arm or the CF+trastuzumab arm. In the study herein, FOLFOX+trastuzumab provided a numerically high OS benefit and statistically significant high PFS benefit. Moreover, although numerically better ORR and disease control rates were obtained in the FOLFOX+trastuzumab arm in the current study, this did not reach statistical significance.

In the study of Gürbüz et al., in which they evaluated maintenance herceptin treatment after chemotherapy in HER2-positive, unresectable, locally advanced or metastatic gastric adenocarcinoma patients, 24 patients received CF+trastuzumab, and 11 patients received mFOLFOX+trastuzumab treatment. All of the patients received a median of 13 maintenance cycles of trastuzumab, with or without capecitabine, after backbone chemotherapy. The median OS was 17.4 months and the median PFS was 12 months. CR was detected in 22% of the patients, PR was detected in 68%, and SD was detected in 8%. In our study, there were 101 patients who continued maintenance trastuzumab after chemotherapy+trastuzumab. The CR rate of the patients was 12.9%, PR rate was 69.3%, and SD rate was 13.9%. The median OS was 23.3 months, while the median PFS was 13.3 (11.6-14.9) months. In both studies evaluating maintenance trastuzumab therapy, OS and PFS seemed to be better in the patients who received maintenance trastuzumab therapy than in the overall patient group. The better response of a group of patients to trastuzumab in the HER2-positive metastatic gastric or GEJ cancer group may be explained by the presence of a different underlying mechanism that increases the response to trastuzumab, other than HER2 positivity. This suggests the difference in tumor biology.

In this study, the rate of discontinuation of treatment due to trastuzumab-related toxicity was 3.3% (8 patients). Treatment was discontinued in 2.8% of the patients due to a low LVEF of grade 3 and above. In the HERXO trial [19], grade 3 and above side effects were observed in 44.4% of patients, asymptomatic LVEF decrease of grade 2 was observed in 2 patients (4.4%), and clinically proven heart failure developed in 1 patient. In another study on patients with metastatic gastric GEJ cancer receiving XELOX+trastuzumab, trastuzumab was discontinued in 6 patients because asymptomatic LVEF decreased below 50%, but no heart failure was reported [21]. In the NSABP-B31 study [23], conducted in breast cancer patients, the rate of development of grade 3-4 heart failure was 1.3% in the chemotherapy (anthracycline and taxane-based chemotherapy)+trastuzumab arm and 4% in the chemotherapy arm. Similarly, in the HERA study [24], conducted in breast cancer patients, a significant LVEF reduction of 4.1% was observed in the arm that received adjuvant trastuzumab for 1 year. It is not possible to conduct a one-to-one comparison because the cytotoxic chemotherapies regimens and the patient population are different in breast cancer and gastric cancer studies, but care should be taken in terms of LVEF reduction and heart failure in patients treated with trastuzumab.

Although there are studies comparing FOLFOX and DCF (docetaxel, cisplatin, and fluorouracil) in terms of the side effect profile in metastatic gastric cancer, there is no large-scale study comparing FOLFOX+trastuzumab with CF+trastuzumab. In a study comparing mDCF and FOLFOX-4 in advanced stage gastric adenocarcinoma, hematological side effects (neutropenia, neutropenic fever, and thrombocytopenia) were more common in the mDCF arm, but neuropathy was seen more frequently in the FOLFOX-4 arm [25]. In the REAL2 trial, triplet therapy with epirubicin and cisplatin+fluorouracil (ECF) or capecitabine (ECX), or triplet therapy with epirubicin and oxaliplatin+fluorouracil (EOF) or capecitabine (EOX) treatment were compared [26]. When compared with cisplatin, oxaliplatin was associated with significantly less grade 3 and higher neutropenia and alopecia but significantly more grade 3 and higher diarrhea and peripheral neuropathy. The phase 3 KEYNOTE-811 study will evaluate the efficacy and safety of pembrolizumab in combination with trastuzumab and chemotherapy as first-line therapy in patients with advanced HER2-positive gastric or GEJ adenocarcinoma. In a study comparing FOLFOX+trastuzumab with CF+trastuzumab, grade 3-4 diarrhea was seen in 2.5% of the CF+trastuzumab arm and 9.3% in the FOLFOX+trastuzumab arm; grade 3-4 thrombosis was seen in 8.6% of the CF+trastuzumab arm and 3.5% in the FOLFOX+trastuzumab arm; and grade 3-4 stomatitis was seen in 11% of the CF+trastuzumab arm and 9% in the FOLFOX+trastuzumab arm [22]. Considering the side effects mentioned in these studies, it can be decided whether the treatment to be added to trastuzumab in HER2-positive patients is FOLFOX or CF.

In this study, there were 23 patients with ECOG PS 2. Patients with poor PS (e.g., ECOG PS >2) are known to generally tolerate chemotherapy poorly, regardless of age, but ECOG PS usually does not adequately reflect the degree of functional impairment in patients [27].

Both in the TOGA trial [18] and HERXO trial [19], after six cycles of chemotherapy+trastuzumab, chemotherapy was stopped and trastuzumab treatment continued until progression or adverse toxicity occurred. However, it may be possible to prolong the duration of chemotherapy in addition to trastuzumab in patients who have not developed chemotherapy toxicity and have a high tumor burden.

There were some limitations in this study. First, it was a retrospective study. Second, metastatic patients were included in the study, but information about the metastasis sites and the number of metastases could not be obtained. Third and one of the most important limitations was the unavailability of the chemotherapy side-effect profiles of the treatments from the hospital records. Therefore, no comparison could be made between the two groups in terms of the chemotherapy side-effect profile.

## CONCLUSION

In this study, mFOLFOX+trastuzumab was similar to CF+trastuzumab in terms of OS and it was more effective in terms of PFS in the first-line treatment of HER2-positive metastatic gastric and GEJ cancer patients. The choice of treatment should be made by considering the prominent toxicity findings of the chemotherapy regimens. However, there are still questions regarding the continuation of chemotherapy with trastuzumab in maintenance therapy in patients who do not have any signs of chemotherapy toxicity and respond to treatment. The fact that the median OS and median PFS were better in the patients receiving maintenance trastuzumab suggested underlying HER2 positivity in this patient group, as well as different tumor biology, which will increase the response to anti-HER2 therapy. Further studies are needed to determine the answers to these questions.

## References

[1] Sitarz R, Skierucha M, Mielko J, Offerhaus GJ, Maciejewski R, Polkowski WP (2018). Gastric cancer:Epidemiology, prevention, classification, and treatment. Cancer Manag Res.

[2] Crew KD, Neugut AI (2006). Epidemiology of gastric cancer. World J Gastroenterol.

[3] Torre LA, Siegel RL, Ward EM, Jemal A (2016). Global cancer incidence and mortality rates and trends--an update. Cancer Epidemiol Biomarkers Prev.

[4] Bray F, Ferlay J, Soerjomataram I, Siegel RL, Torre LA, Jemal A (2018). Global cancer statistics 2018:GLOBOCAN estimates of incidence and mortality worldwide for 36 acancers in 185 countries. CA Cancer J Clin.

[5] Karunagaran D, Tzahar E, Beerli RR, Chen X, Graus-Porta D, Ratzkin BJ (1996). ErbB-2 ais a common auxiliary subunit of NDF and EGF receptors:Implications for breast cancer. Embo J.

[6] Petit AM, Rak J, Hung MC, Rockwell P, Goldstein N, Fendly B (1997). Neutralizing antibodies against epidermal growth factor and ErbB-2/neu receptor tyrosine kinases down-regulate vascular endothelial growth factor production by tumor cells *in vitro* and *in vivo*:Angiogenic implications for signal transduction therapy of solid tumors. Am J Pathol.

[7] Giatromanolaki A, Koukourakis MI, Simopoulos C, Polychronidis A, Gatter KC, Harris AL (2004). c-erbB-2 arelated aggressiveness in breast cancer is hypoxia inducible factor-1alpha dependent. Clin Cancer Res.

[8] Slamon DJ, Clark GM, Wong SG, Levin WJ, Ullrich A, McGuire WL (1987). Human breast cancer:Correlation of relapse and survival with amplification of the HER-2/neu oncogene. Science.

[9] Swain SM, Baselga J, Kim SB, Ro J, Semiglazov V, Campone M (2015). Pertuzumab, trastuzumab, and docetaxel in HER2-positive metastatic breast cancer. N Engl J Med.

[10] Chua TC, Merrett ND (2012). Clinicopathologic factors associated with HER2-positive gastric cancer and its impact on survival outcomes--a systematic review. Int J Cancer.

[11] Gómez-Martin C, Garralda E, Echarri MJ, Ballesteros A, Arcediano A, Rodríguez-Peralto JL (2012). HER2/neu testing for anti-HER2-based therapies in patients with unresectable and/or metastatic gastric cancer. J Clin Pathol.

[12] Janjigian YY, Werner D, Pauligk C, Steinmetz K, Kelsen DP, Jäger E (2012). Prognosis of metastatic gastric and gastroesophageal junction cancer by HER2 astatus:A European and USA international collaborative analysis. Ann Oncol.

[13] Tanner M, Hollmén M, Junttila TT, Kapanen AI, Tommola S, Soini Y (2005). Amplification of HER-2 ain gastric carcinoma:Association with Topoisomerase IIalpha gene amplification, intestinal type, poor prognosis and sensitivity to trastuzumab. Ann Oncol.

[14] Yan B, Yau EX, Omar SS, Ong CW, Pang B, Yeoh KG (2010). A study of HER2 agene amplification and protein expression in gastric cancer. J Clin Pathol.

[15] Grabsch H, Sivakumar S, Gray S, Gabbert HE, Müller W (2010). HER2 aexpression in gastric cancer:Rare heterogeneous and of no prognostic value-conclusions from 924 cases of two independent series. Cell Oncol.

[16] Gravalos C, Jimeno A (2008). HER2 ain gastric cancer:A new prognostic factor and a novel therapeutic target. Ann Oncol.

[17] NCCN Clinical Practice Guidelines in Oncology (2022). Gastric Cancer, Version 2.2022.

[18] Bang YJ, Van Cutsem E, Feyereislova A, Chung HC, Shen L, Sawaki A (2010). Trastuzumab in combination with chemotherapy versus chemotherapy alone for treatment of HER2-positive advanced gastric or gastro-oesophageal junction cancer (ToGA):A phase 3, open-label, randomised controlled trial. Lancet.

[19] Rivera F, Romero C, Jimenez-Fonseca P, Izquierdo-Manuel M, Salud A, Martinez E (2019). Phase II study to evaluate the efficacy of Trastuzumab in combination with capecitabine and oxaliplatin in first-line treatment of HER2-positive advanced gastric cancer:HERXO trial. Cancer Chemother Pharmacol.

[20] Ryu MH, Yoo C, Kim JG, Ryoo BY, Park YS, Park SR (2015). Multicenter phase II study of trastuzumab in combination with capecitabine and oxaliplatin for advanced gastric cancer. Eur J Cancer.

[21] Gong J, Liu T, Fan Q, Bai L, Bi F, Qin S (2016). Optimal regimen of trastuzumab in combination with oxaliplatin/capecitabine in first-line treatment of HER2-positive advanced gastric cancer (CGOG1001):A multicenter, phase II trial. BMC Cancer.

[22] Jimenez-Fonseca P, Carmona-Bayonas A, Martinez-Torron A, Alsina M, Custodio A, Serra O (2021). External validity of clinical trials with diverse trastuzumab-based chemotherapy regimens in advanced gastroesophageal adenocarcinoma:Data from the AGAMENON-SEOM registry. Ther Adv Med Oncol.

[23] Advani PP, Ballman KV, Dockter TJ, Colon-Otero G, Perez EA (2016). Long-term cardiac safety analysis of NCCTG N9831 (alliance) adjuvant trastuzumab trial. J Clin Oncol.

[24] de Azambuja E, Procter MJ, van Veldhuisen DJ, Agbor-Tarh D, Metzger-Filho O, Steinseifer J (2014). Trastuzumab-associated cardiac events at 8 ayears of median follow-up in the herceptin adjuvant trial (BIG 1-01). J Clin Oncol.

[25] Pourghasemian M, Danandeh Mehr A, Molaei M, Habibzadeh A (2020). Outcome of FOLFOX and modified DCF chemotherapy regimen in patients with advanced gastric adenocarcinoma. Asian Pac J Cancer Prev.

[26] Cunningham D, Starling N, Rao S, Iveson T, Nicolson M, Coxon F (2008). Capecitabine and oxaliplatin for advanced esophagogastric cancer. N Engl J Med.

[27] Rodin MB, Mohile SG (2007). A practical approach to geriatric assessment in oncology. J Clin Oncol.

